# The Role of miR‐144 in Inflammatory Diseases: A Review

**DOI:** 10.1002/iid3.70172

**Published:** 2025-03-11

**Authors:** Shukun Hong, Hongye Wang, Lujun Qiao

**Affiliations:** ^1^ Department of Intensive Care Unit Shengli Oilfield Central Hospital Dongying Shandong China; ^2^ Clinical Research Center of Dongying Critical Care Medicine Dongying Shandong China; ^3^ Department of Obstetrics and Gynecology Shengli Oilfield Central Hospital Dongying Shandong China

**Keywords:** biomarkers, inflammation, microRNA, miR‐144, review, targeted treatment

## Abstract

**Background:**

Inflammation, often caused by various stimuli, is a common response to tissue homeostasis disruptions and is considered a key driver of many pathological conditions. MicroRNA‐144 (miR‐144) has emerged as a critical regulator in inflammatory diseases, with its dysregulation implicated in various pathological conditions. Understanding its role and mechanisms is essential for developing therapeutic strategies.

**Objective:**

This article aimed to evaluate the role of miR‐144 in inflammatory diseases through a literature review.

**Methods:**

Electronic databases including PubMed, Web of Science, Springer Link, China Knowledge Resource Integrated Database, and Wanfang Data were searched for relevant literature. The following keywords were used and combined differently according to the rules of the databases: “miR‐144,” “inflammation,” “inflammatory,” and “immune response.” Studies investigating miR‐144 in the context of inflammation were included. Data were extracted to assess miR‐144's expression patterns and its association with disease severity and outcomes.

**Results:**

miR‐144 was found to be differentially expressed in a range of inflammatory diseases, including sepsis, infectious diseases, respiratory diseases, cardiovascular diseases, digestive diseases, neuropsychiatric diseases, arthritis, and pregnancy complications. The expression patterns varied depending on the disease, with both upregulation and downregulation observed. miR‐144 was implicated in the modulation of inflammatory responses through direct and indirect targeting of key proteins and pathways. The review also highlighted the potential of miR‐144 as a diagnostic and prognostic biomarker.

**Conclusion:**

miR‐144 plays a significant role in the pathogenesis of inflammatory diseases and holds promise as a biomarker. Its expression patterns and regulatory mechanisms offer insights into disease processes and may guide future therapeutic strategies. However, further clinical studies are needed to validate miR‐144's utility as a biomarker and to explore its therapeutic potential in a clinical setting.

## Graphical abstract

In most cases, the expression of miR‐144 was downregulated, and it exerted corresponding biological effects by negatively regulating downstream target genes. Our reviews show that miR‐144 has the potential to become a diagnostic biomarker and therapeutic target for various inflammatory diseases.

## 1 Introduction

Inflammation is a common response to disruptions in tissue homeostasis, often triggered by various stimuli such as pathogen infections, ischemic injuries, trauma, toxin exposure, or abnormal substances that activate the innate and adaptive immune responses. In the past few decades, inflammation has been extensively researched and is recognized as a key driving factor in many pathological conditions [1]. If the inflammatory trigger persists or the body's control mechanisms fail, inflammation may become chronic [2]. Chronic inflammation is linked to a range of diseases, including cardiovascular disease, cancer, diabetes, arthritis, Alzheimer's disease, lung diseases, and autoimmune diseases. Therefore, identifying and regulating key factors in inflammatory mechanisms is crucial for preventing and managing inflammation‐related diseases.

With advances in genetic testing technology, the key role of microRNAs (miRNAs) in inflammation has become increasingly apparent. MiRNAs are small, non‐coding RNA molecules composed of 18–25 nucleotides that can bind to the 3'‐untranslated region (UTR) of target messenger RNA (mRNA), regulating the expression of multiple protein‐coding genes through post‐transcriptional mechanisms such as translational inhibition or mRNA degradation [3]. Under normal conditions, miRNAs are involved in physiological processes likecell differentiation, proliferation, apoptosis, hematopoiesis, and metabolism. However, during pathological conditions characterized by disrupted homeostasis, the abnormal expression of miRNAs can contribute to disease states by regulating multiple genes [2]. There is growing evidence that miR‐144, in particular, acts as an important regulator of the inflammatory response and is implicated in the development of inflammation‐related diseases, such as sepsis [4], pulmonary tuberculosis (PTB) [5], arthritis [6], and tumors [7]. Understanding the expression and mechanisms of miR‐144 in these diseases can aid in their diagnose and treatment.

In this paper, we described the biosynthesis of miR‐144, summarized its expression across various inflammatory diseases, and discussed the mechanisms by which it is involved in disease onset and progression. We also explored the potential clinical applications of miR‐144.

## 2 Literature Search

To comprehensively collect relevant literature for this review, the following electronic databases were searched: PubMed, Web of Science, Springer Link, China Knowledge Resource Integrated Database, and Wanfang Data. The following keywords were used and combined differently according to the rules of the databases: “miR‐144,” “inflammation,” “inflammatory,” “immune response.” Prospective and retrospective clinical studies, both comparative and non‐comparative; observational studies include animal and molecular biology experiments; narrative reviews; and meta‐analysis were included. Articles with insufficient data, including case reports, letters, editorials, and comments, were excluded. All references cited in the included articles were screened to identify relevant articles. All stages of literature search, study selection, and data extraction were performed by two authors (S.H., H.W.).

## 3 Biosynthesis of miR‐144

**Figure 1:**
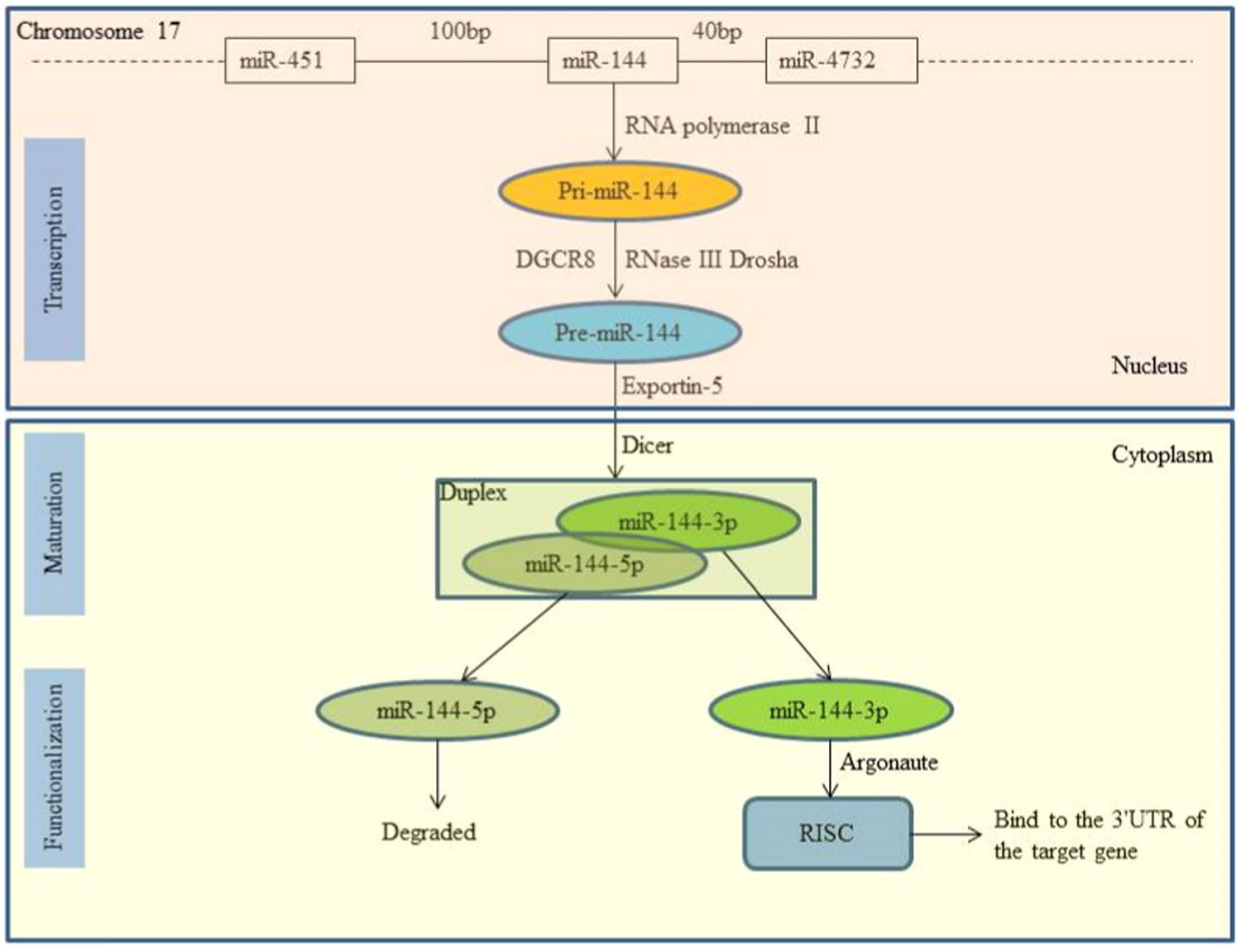
The biosynthesis process of miR‐144 in cells.

Like other miRNAs, miR‐144 undergoes typical miRNA biogenesis involving transcription, maturation, and functionalization (Figure 1). Its gene is located on chromosome 17q11.2, where RNA polymerase II transcribes it into a primary transcript, pri‐miR‐144 [8]. Within the nucleus, Drosha and DGCR8 convert pri‐miR‐144 into premiR‐144, which is then exported to the cytoplasm by Exportin‐5 [9]. Dicer further processes premiR‐144 into a mature miRNA duplex consisting of a mature miRNA (guide strand, miR‐144‐3p) and its antisense strand (passenger strand, miR‐144‐5p) [10]. Only miR‐144‐3p, being thermodynamically stable, forms the RNA‐induced silencing complex (RISC) with Argonaute proteins to regulate gene expression by binding to the 3′‐UTR of the target gene [11].

## 4 miR‐144 and Sepsis

Sepsis, a life‐threatening organ dysfunction, results from a dysregulated host response to infection. It is characterized by a combination of shock, acute lung injury (ALI), and acute kidney injury (AKI) [12]. Numerous studies have established a mutual relationship between miRNAs and sepsis [13, 14, 15]. Qin et al. [16] recruited 3 sepsis patients and 3 healthy subjects to identify key genes and miRNAs related to sepsis and conducted RNA sequencing and bioinformatics analysis. The study identified 1199 differentially expressed mRNAs (DEmRNAs) and 23 differentially expressed miRNAs (DEmiRNAs), with miR‐144‐3p being among the top three DEmiRNAs covering most DEmRNAs. Ahmad et al. [17] analyzed miRNA expression data from 15 blood samples (six from patients with sepsis combined with AKI, six from patients with sepsis combined with non‐AKI, and three from healthy subjects) using a computer network method. The results revealed that 317 sepsis‐related genes were regulated by 10 DEmiRNAs, one of which, miR‐144‐3p, was downregulated. Song et al. [18] also detected decreased levels of miR‐144‐3p in the blood of newborns with sepsis. These studies all suggest a close relationship between miR‐144 and the occurrence and development of sepsis.

Schmidt and colleagues [19] observed changes in miR‐144‐3p expression in the aorta of septic mice, with hypoxia‐inducible factor‐1 alpha (HIF‐1α) identified as a target, playing a significant role in the process of angiogenesis [20]. It has been reported that miR‐144‐3p was downregulated in lipopolysaccharide (LPS)‐treated cardiomyocytes and was involved in regulating the nuclear factor‐kappa B (NF‐κB) signaling pathway [4]. The overexpression of miR‐144‐3p enhanced the viability of injured cardiomyocytes, inhibited cell apoptosis, and alleviated the inflammatory response. Rho‐related kinase‐1 (ROCK1) is a serine/threonine kinase that plays a vital role in the pathogenesis of many inflammatory diseases, including atherosclerosis, diabetes, and ALI [21, 22, 23, 24]. Siddiqui et al. [25] reported that miR‐144‐mediated inhibition of ROCK1 could reduce the permeability of LPS‐induced pulmonary endothelial cells.

All the aforementioned research reports indicate that the role of miR‐144 in sepsis is achieved through the downregulation of its expression. However, contrary findings have also been observed. Tod et al. [26] found that the expression of miR‐144‐3p was upregulated in the kidneys of septic AKI mice. This upregulation was also observed by Xu et al. [27] in animal models of septic ALI. This study also showed that LPS‐induced inflammation and cell apoptosis could be mitigated by the miR‐144‐3p antagomir and exacerbated by the miR‐144‐3p agomir or sh‐Caveolin‐2 treatment. Furthermore, researchers discovered that miR‐144‐3p could activate the Janus kinase/signal transducer and activator of transcription (JAK/STAT) signaling pathway by downregulating Caveolin‐2 expression, thereby promoting LPS‐induced ALI. In addition, upregulated miR‐144‐3p was shown to negatively regulate its target aquaporin‐1 [28], a factor closely linked to the development of ALI [29]. The diverse roles of miR‐144 in sepsis, as well as its interaction with related signaling pathways, offer new insights for the early diagnosis and treatment of sepsis.

## 5 miR‐144 and Infectious Diseases

### 5.1 Bacterial Infection

Due to its role in regulating sepsis, the value of miR‐144 in the immune response to microbial infections, such as bacteria and viruses, is increasingly recognized. Kim et al. [30] observed the upregulation of miR‐144‐3p and proinflammatory cytokines/chemokines in peripheral blood mononuclear cells of patients infected with *Mycobacteroides abscessus*. Moreover, they found a strong correlation between miR‐144‐3p expression and these inflammatory markers, highlighting its importance in pathological inflammation during *Mycobacteroides abscessus* infection.

It is believed that miR‐144 may serve as a biomarker for the clinical diagnosis of active PTB. Zhou et al. [31] found that the expression levels of inflammatory factors, including tumor necrosis factor‐alpha (TNF‐α), interleukin (IL)‐10, and monocyte chemoattractant protein‐1 (MCP‐1), were significantly elevated in the plasma of patients with active PTB compared to healthy volunteers. In contrast, the expression level of miR‐144 was significantly reduced, indicating a negative correlation. By plotting receiver operating characteristic (ROC) curves, the sensitivity and specificity of using miR‐144 to diagnose active PTB were found to be as high as 96.88% and 96.77%, respectively. Liu [5] observed the downregulation of miR‐144 in monocyte‐derived macrophages infected with *Mycobacterium tuberculosis*. Inhibition of miR‐144 activated the extracellular signal‐regulated kinase (ERK) signaling pathway by inducing ERK1/2 phosphorylation and simultaneously accelerated the secretion of TNF‐α, IL‐1β, and IL‐6. However, the expression level of miR‐144 does not consistently decrease after infection with *Mycobacterium tuberculosis*. A recent meta‐analysis, which included nine articles, revealed a significant increase in serum miR‐144 levels in PTB patients compared to healthy individuals [32]. The discrepancies in the findings between the two studies could be partially attributed to the differences in sample sources. In the context of infection, macrophages are known to potentially modulate the expression of miR‐144 through diverse molecular mechanisms, while the levels of serum miRNA may be subject to influence from various cell types or systemic inflammatory responses. In addition, the differences in infection conditions, study designs, individual variability, and the potential impact of inflammatory states or complications associated with PTB may also contribute to differences in results. To better understand this paradoxical phenomenon, further research is needed to explore the role of miR‐144 in PTB and the changes in miR‐144 expression levels under different sample types and infection conditions.

### 5.2 Virus Infection

Recent research has unveiled some key findings regarding the role of miR‐144 in viral infections. Visacri et al. [33] analyzed 20 studies related to COVID‐19, identifying miR‐144 as one of the five most important dysregulated miRNAs. This finding suggests its potential as a biomarker for diagnosing COVID‐19 and assessing its severity. In another study, Rosenberger et al. [34] observed that the ectopic expression of miR‐144 in primary mouse lung epithelial cells could enhance the replication of the influenza virus, myocarditis virus, and vesicular stomatitis virus. Moreover, the ablation of miR‐144 in vivo could reduce the replication of influenza virus in the lungs and the disease severity. TNF receptor‐associated factor 6 (TRAF6) and interferon regulatory factor 7 (IRF7) are known to play crucial roles in immune responses to pathogens [35, 36]. Rosenberger et al. [34] also demonstrated that miR‐144 could inhibit TRAF6 expression and IRF7 activity, revealing that miR‐144 may reduce the host's immune response to the influenza virus by disrupting the TRAF6‐IRF7 pathway.

## 6 miR‐144 and Respiratory Diseases

### 6.1 Chronic Obstructive Pulmonary Disease (COPD)

COPD is a common chronic inflammatory respiratory disease often linked to long‐term smoking [37]. It has been reported that the dysregulation of miRNAs can affect the occurrence and progression of COPD [38]. In 16HBE human bronchial epithelial cells exposed to cigarette smoke, there is an increase in proinflammatory factors such as IL‐1β, IL‐6, IL‐8, and TNF‐α, alongside an upregulation of miR‐144 [39, 40]. miR‐144 in 16HBE cells has been confirmed to target PH domain leucine‐rich repeat protein phosphatase 2 (PHLPP2) [40], which in turn promotes protein kinase B (Akt) phosphorylation and the production of inflammatory factors [41]. This finding suggests that the miR‐144/PHLPP2/Akt axis is involved in COPD pathogenesis. Further studies have confirmed the upregulation of miR‐144 in COPD patients [42]. Additionally, a decrease in enhancer of zeste homolog 2 (EZH2), a histone methyltransferase, was observed in COPD patients and inflammatory cell models. Luciferase reporter assays (LRA) showed that EZH2 is a downstream target of miR‐144‐3p, which negatively regulates it. This implies that the miR‐144‐3p/EZH2 axis may contribute to COPD progression.

### 6.2 Lung Cancer

Chronic inflammation and infection are key factors in cancer development [43]. miRNAs, as novel inflammatory molecules, are involved in various stages of tumor development, including initiation, progression, invasion, and metastasis [44, 45, 46]. A study [7] identified IL‐1β and miR‐144‐3p as independent risk factors for lung adenocarcinoma (LUAD). LUAD patients exhibit higher plasma IL‐1β levels and lower miR‐144‐3p expression compared to healthy individuals, indicating an inverse correlation. In vitro, IL‐1β stimulation reduced miR‐144‐3p expression in A549 cells. Moreover, IL‐1β and miR‐144‐3p inhibitors enhanced A549 cell proliferation, while miR‐144‐3p mimics attenuated the proliferative effect of IL‐1β. Subsequent research [47] identified Wilms tumor 1 (WT1) as a downstream target of miR‐144‐3p, whose expression is negatively regulated by miR‐144‐3p. These findings suggest that the IL‐1β/miR‐144‐3p/WT1 axis may mediate LUAD development through inflammatory processes. Additionally, elevated Toll‐like receptor 2 (TLR2) levels in non‐small cell lung cancer tissues and cell lines are negatively regulated by miR‐144, which could reduce the secretion of IL‐1β, IL‐6, and IL‐8 from A549 cells and inhibit their migration and invasion [48]. These findings provide a theoretical foundation for considering miR‐144 as a novel therapeutic target for lung cancer.

### 6.3 Asthma

As a component of the epigenetic regulation machinery, miR‐144 has been linked to specific disease features in asthma [49, 50]. Rodrigo‐Munoz et al. [51] assessed the levels of miR‐144‐3p in airway biopsies and serum from asthmatics and healthy individuals. The research team observed that miR‐144‐3p was increased in asthmatic lungs, and its presence correlated directly with blood eosinophilia and with the expression of genes involved in asthma pathophysiology in the airways. The study also showed that serum miR‐144‐3p was increased in patients with severe asthma and was associated with higher doses of corticosteroid treatment. Overexpression of miR‐144‐3p in bronchial smooth muscle cells could affect the expression of the transcription factor GATA3, which plays a vital role in eosinophilia associated with asthma [52]. Another study [53] revealed that increased serum miR‐144‐3p expression was closely related to increased use of short‐acting β2‐agonists in children with asthma. These studies highlight the roles and biomarker potential of miR‐144 as a biomarker for asthma severity. Additionally, miR‐144 has been implicated in the regulation of airway epithelial cell functions, which are crucial in the pathogenesis of asthma [54]. The regulation of miR‐144 through epigenetic mechanisms underscores its importance in the context of asthma, where environmental factors and genetic predispositions interact to influence disease outcomes.

## 7 miR‐144 and Cardiovascular Diseases

### 7.1 Myocardial Ischemia

In the context of cardiovascular health, the interplay between myocardial ischemia and inflammation is a critical area of focus. Zhang and colleagues [55] reported that the dissolution of the chromatin remodeling factor Brg1 in endothelial cells of the human heart microvasculature could suppress the infiltration of neutrophils and decrease the levels of proinflammatory mediators such as IL‐1, IL‐6, TNF‐α, and monocyte chemotactic protein 1 after ischemia‐reperfusion injury. As confirmed by previous research [56], miR‐144 can negatively regulate Brg1 expression, while the long‐chain noncoding RNA (lncRNA) MALAT1 can inhibit miR‐144 function [57]. This suggests that the MALAT1/miR‐144/Brg1 axis may be an inflammatory regulation pathway in myocardial ischemia‐reperfusion injury [58]. Additionally, NOD‐like receptor protein 3 (NLRP3) plays a key role in myocardial ischemia‐reperfusion injury [59]. Yang et al. [60] discovered that the level of miR‐144‐3p in cardiac cells after ischemia‐reperfusion injury was reduced, and upregulating miR‐144‐3p attenuated this type of heart damage. Further exploration revealed that miR‐144‐3p could inhibit the activation of the NLRP3 inflammasome in cardiac cells by targeting calcium‐activated chloride channel protein 1 (Anoctamin 1, ANO1). This study suggests that the miR‐144‐3p/ANO1 axis may be a new therapeutic target for myocardial ischemia.

### 7.2 Atherosclerosis

Chronic inflammation is involved in the development of atherosclerosis [61]. Hu et al. [62] reported elevated plasma levels of miR‐144‐3p in patients with acute myocardial infarction, positively correlated with changes in cardiac enzymes. Through in vitro and in vivo experiments, they found that miR‐144‐3p mimics could increase the release of proinflammatory factors (IL‐1β, IL‐6, and TNF‐α) and target ATP binding cassette transporter A1 (ABCA1), a key protein in extrahepatic reverse cholesterol transport, thereby inhibiting its expression, blocking reverse cholesterol transport, reducing plasma high‐density lipoprotein levels, and accelerating atherosclerotic progression in mice. This supports considering miR‐144‐3p as a therapeutic target for atherosclerosis and a biomarker for acute myocardial infarction.

### 7.3 Abdominal Aortic Aneurysms (AAA)

Evidence shows that the disruption of miRNAs contributes to the formation of AAA [63, 64]. Shi et al. [65] reported reduced expression of miR‐144‐5p in AAA tissue. Further research showed that miR‐144‐5p mimics could attenuate aortic expansion and elastic degradation induced by angiotensin II in *ApoE* ^*−/−*^ mice, thereby reducing AAA incidence and improving survival rates. In addition, the study identified TLR2 and oxidized low‐density lipoprotein receptor 1 (OLR1) as targets of miR‐144‐5p, both negatively regulated by it. miR‐144‐5p mimics could also suppress the upregulation of M1 macrophage inflammatory markers induced by oxidized low‐density lipoprotein and inhibit downstream signaling pathways of TLR2 and OLR1, such as NF‐κB and ERK1/2, involved in AAA development. This study indicates that miR‐144‐5p may protect against AAA through its effect on macrophage‐related inflammation.

## 8 miR‐144 and Digestive Diseases

### 8.1 Non‐Alcoholic Steatohepatitis

Studies have indicated that miRNAs play a significant role in regulating the chronic inflammation associated with steatohepatitis. Liu et al. [66] reported a decrease in miR‐144‐5p expression in patients with non‐alcoholic fatty liver disease. Similarly, Li et al. [67] noticed a negative correlation between miR‐144 expression and TLR2 in Kupffer cells from nonalcoholic steatohepatitis rats. Treatment with miR‐144 mimics reduced the activation of TLR2, TNF‐α, and NF‐κB in rat macrophage lines NR8383 and BMM, while miR‐144 inhibitors exhibited the opposite effect.

### 8.2 Inflammatory Bowel Disease (IBD)

Given the close relationship between IBD and immune system dysfunction, miRNA plays a role in the pathogenesis of IBD by its ability to influence immune responses [68]. Recently, there are reports that the upregulation of miR‐144 is closely related to Crohn's disease [69]. De ludicibus et al. [70] found that miR‐144 expression was upregulated in the peripheral blood of children with IBD who responded to glucocorticoid therapy after 4 weeks, speculating that its target may be the 3′‐UTR of the glucocorticoid receptor gene. This study indicates that miR‐144 could serve as a pharmacogenomic biomarker for the response of IBD patients to glucocorticoids.

Ginsenoside RT4 (RT4) is a novel bioactive compound extracted from ginseng, which possesses various pharmacological properties [71]. Li et al. [72] discovered that RT4 could effectively mitigate the colonic damage in ulcerative colitis mice induced with dextran sulfate sodium, reduce TNF‐α and IL‐1β levels, and increase the expression of the anti‐inflammatory cytokine IL‐10. In‐depth research revealed that RT4 upregulated the expression of miR‐144‐3p in colitis mice. LRA confirmed that the solute carrier family 7 member 11 (SLC7A11) was the target gene of miR‐144‐3p. Moreover, RT4 could inhibit the activation of the miR‐144‐3p/SLC7A11 signaling pathway, thereby alleviating the progression of colitis.

### 8.3 Other Digestive Diseases

Evidence indicates that miR‐144 can regulate the expression of nuclear factor E2‐related factor 2 (Nrf2), which has antioxidant stress and anti‐inflammatory response functions [1, 73]. Lin et al. [74] observed that the antagonists of miR‐144 could increase Nrf2 expression in the esophageal mucosa of rats with reflux esophagitis, thereby reducing inflammation and preserving the esophageal barrier function. Han et al. [75] detected a significant downregulation of miR‐144 expression in the serum and tumor samples of colorectal cancer patients, however, the expression of C‐X‐C motif chemokine ligand 11 (CXCL11), a regulator of chronic inflammation, was significantly increased [76, 77]. LRA showed that miR‐144 directly targets the 3′‐UTR of the CXCL11 mRNA to regulate its expression. These findings suggest that miR‐144 can mediate chronic inflammation during the progression of colorectal cancer by regulating CXCL11.

## 9 miR‐144 and Neuropsychiatric Diseases

### 9.1 Neuropathic Pain

The anti‐inflammatory role of miR‐144 in neurological diseases has garnered increasing attention. Zhang et al. [78] discovered that miR‐144 expression was significantly reduced in mice with neuropathic pain induced by chronic constriction injury. Intrathecal injection with a miR‐144 agonist alleviated mechanical and heat pain hypersensitivity in these animals. miR‐144 has also been shown to negatively regulate neuroinflammation by decreasing the expression of proinflammatory mediators such as IL‐1β, IL‐6, and TNF‐α, which is beneficial for inhibiting the development of neuropathic pain. Further research revealed that Ras p21 protein activator 1 (RASA1), a target of miR‐144, can reverse the analgesic effect of miR‐144 when overexpressed, suggesting its potential as a therapeutic target for neuropathic pain.

### 9.2 Stroke

Stroke is intricately linked to inflammation, as the immune response can exacerbate neuronal damage and contribute to the progression of the disease. Wang et al. [79] reported that knockdown of the miR‐144/451 cluster in collagenase‐induced cerebral hemorrhage mice enhanced the secretion of TNF‐α and IL‐1β, as well as oxidative stress in the perihematomal region. This resulted in exacerbated neurological functional deficits and cerebral edema, suggesting a protective role for the miR‐144/451 cluster in cerebral hemorrhage. Li et al. [80] observed upregulation of proinflammatory factors (IL‐1β, TNF‐α, and IL‐6) and downregulation of the anti‐inflammatory factor IL‐10, along with decreased expression of miR‐144‐5p, in the brain tissue of rats with focal cerebral ischemia. Upregulating miR‐144‐5p expression could suppress the inflammatory response in the brain, reduce oxidative stress and neuronal apoptosis, and improve angiogenesis and neural function recovery.

### 9.3 Neurodegenerative Diseases

Xing et al. [81] detected decreased miR‐144 expression and elevated TNF‐α expression in the brains of patients with Parkinson's disease, confirming a negative correlation between them. Transfection with miR‐144 inhibitors promoted the translocation of p65 from the cytoplasm to the nucleus and activated the NF‐κB signaling pathway. This study revealed the role of disrupted miR‐144 in Parkinson's disease pathogenesis.

Oxidative stress and proinflammatory changes in cerebral vascular endothelial cells are known to promote aging, with Nrf2 playing a crucial role in vascular protection and aging regulation through the coordination of cellular responses to oxidative stress [82, 83]. Evidence suggests that dysregulated miRNAs are also involved in the aging process [84, 85]. Csiszar et al. [86] found that miR‐144 expression was upregulated in the microvascular endothelial cells of older rats, and overexpression of miR‐144 reduced Nrf2 expression. Overexpression of a miR‐144 antagonist significantly increased Nrf2 levels, exerting anti‐aging effects through antioxidant and anti‐inflammatory pathways.

### 9.4 Depression

In recent years, the role of miRNAs in depression has been a growing area of interest. Lower levels of circulating miR‐144‐5p are related to depression and anxiety, with significant changes observed after psychological therapy [87]. Sundquist et al. found that in patients who received 8 weeks of psychological treatment for depression, anxiety, or stress and adjustment disorders, the levels of 21 different inflammatory proteins tended to decrease, corresponding with an increase in miR‐144‐5p post‐treatment [88]. Based on these reports, it is proposed that miR‐144 may contribute to the development of mental diseases by regulating components of the proinflammatory signaling pathway.

## 10 miR‐144 and Arthritis

### 10.1 Rheumatoid Arthritis (RA)

RA is a systemic, chronic, and inflammatory disease characterized by chronic synovitis and clinical fluctuations, eventually leading to joint deformity and long‐term disability. Evidence suggests that miRNAs can be abnormally expressed in the blood of RA patients [89]. However, the expression and biological effects of miR‐144 in different RA models are inconsistent. Studies [90, 91] have shown that the expression of miR‐144‐3p is upregulated in IL‐1β‐stimulated N1511 chondrocytes. The absence of miR‐144‐3p increases cell viability, inhibits apoptosis, reduces the release of proinflammatory factors such as IL‐1β and TNF‐α, and decreases the loss of extracellular matrix. Moreover, inhibition of miR‐144‐3p has been shown to alleviate cartilage injury and inflammation in RA rats. Additionally, miR‐144‐3p targets and negatively regulates the expression of bone morphogenetic protein 2 (BMP2) or phosphatase and tensin homolog (PTEN), which act on the phosphatidylinositol 3‐kinase (PI3K)/Akt pathway or the PTEN‐induced putative kinase 1 (PINK1)/Parkin pathway, respectively, exacerbating cartilage cell damage and the inflammatory response.

In contrast, Zhou et al. [92] reported that miR‐144‐5p expression was downregulated in THP‐1 macrophages treated with LPS. Overexpression of miR‐144‐5p decreased macrophages viability and reduced the levels of TNF‐α, IL‐6, and IL‐8, as well as the phosphorylation of p65 in the TLR2 and NF‐κB signaling pathways. Silencing TLR2 yielded similar results. In an RA rat model induced by type II collagen, overexpression of miR‐144 specifically downregulated the expression of calcium‐binding protein 11 (CBP11), alleviating the inflammatory response in synovial tissues [93]. In addition, traditional Chinese medicine moxibustion therapy has also been shown to upregulate miR‐144‐3p, which negatively regulates its target HIF‐1α, restores the imbalance of Treg/Th17 cell differentiation, and reduces the levels of proinflammatory factors, offering a potential treatment for RA [94].

### 10.2 Osteoarthritis

Lin et al. [6] reported a negative correlation between miR‐144‐3p and IL‐1β expression in the synovial tissue of osteoarthritis patients. In vitro, transfection of miR‐144‐3p mimics into human articular chondrocytes led to downregulation of IL‐1β expression and inhibition of the PI3K/Akt, NF‐κB, and mitogen‐activated protein kinase (MAPK) signaling pathways related to IL‐1β production. Administration of miR‐144‐3p mimics to an anterior cruciate ligament transection rat model helped alleviate the progression of osteoarthritis and reduce the quantity of IL‐1β‐positive cells in the synovial tissue.

Research indicates that lncRNA plays a role in the development of osteoarthritis. Liu et al. [95] found that knockdown of lncRNA TUG1 promoted apoptosis and inflammation in osteoarthritis chondrocytes. TUG1 was observed to target miR‐144‐3p, which in turn targeted dual‐specificity phosphatase 1 (DUSP1), a key negative regulator of the inflammatory response. TUG1 inhibited apoptosis and inflammation by competitively binding to miR‐144‐3p, enhancing DUSP1 expression and suppressing the p38 MAPK pathway. This study elucidated the role of the TUG1/miR‐144‐3p/DUSP1/p38 MAPK regulatory network in osteoarthritis chondrocyte injury. Yi et al. [96] conducted similar research, finding that miR‐144‐3p expression decreased while lncRNA TM1‐3p and ONECUT2 (a transcription factor) expressions increased in stimulated cells. Overexpression of miR‐144‐3p downregulated ONECUT2 expression, reduced proliferation, and promoted apoptosis in IL‐1β‐induced synoviocytes. Silencing TM1‐3P alleviated the damage of rat knee joint tissue and reduced the levels of IL‐1β, IL‐6, and TNF‐α. LRA confirmed that the upstream target of miR‐144‐3p was TM1‐3P and the downstream target was ONECUT2, suggesting that the TM1‐3P/miR‐144‐3P/ONECUT2 axis improves knee inflammation and injury in osteoarthritis rats.

### 10.3 Other Arthritis

Studies also suggest that miR‐144 may be involved in the pathogenesis of ankylosing spondylitis [97] and inflammatory bone resorption diseases [98], although the exact mechanisms remain unclear.

## 11 miR‐144 and Pregnancy Complications

### 11.1 Premature Labor

Preterm labor, often caused by intraamniotic inflammation, is associated with increased prostaglandin E2 levels in amniotic fluid [99]. Li and colleagues' [100] animal experiments induced by lipopolysaccharide (LPS) demonstrated that LPS stimulation upregulates miR‐144 expression in the amniotic membrane. This study showed that miR‐144 directly targets the oncogene c‐fos and indirectly increases cyclooxygenase‐2 (COX2) expression, leading to elevated prostaglandin E2 levels, uterine smooth muscle contraction, and consequently, premature labor.

### 11.2 Preeclipsia (PE)

PE is a severe hypertensive disorder during pregnancy, characterized by placental hypoxia, inflammation, and oxidative stress [101]. Mesenchymal stem cell‐derived exosomes (Exos‐MSC) have been shown to mitigate inflammatory diseases [102, 103]. Sun et al. [104] observed in PE‐like models that silencing FosB, overexpressing miR‐144, or treating with Exos‐MSC overexpressing miR‐144 (Exos‐MSC^miR‐144^) reversed the LPS‐induced decline in HTR‐8/SVneo cell viability and migration. These treatments also reduced the LPS‐induced increase in IL‐6, TNF‐α, NF‐κB, soluble FMS‐like tyrosine kinase 1 (sFlt‐1), and Flt‐1 levels. Administering Exos‐MSC^miR‐144^ to PE‐like pregnant rats reversed the LPS‐induced increase in FosB expression, inflammatory factors, sFlt‐1 levels, and blood pressure. These findings suggest that miR‐144 can mitigate gestational hypertension and inflammation by regulating the FosB/Flt‐1 pathway, potentially aiding in the development of new therapies for PE.

## 12 miR‐144 and Diabetes

Research indicates that in patients with poorly controlled type 2 diabetes (HbA1c > 8%), plasma concentrations of proinflammatory cytokines such as IL‐6, IL‐8, IL‐18, IL‐23, TNF‐α, and CRP are elevated, correlating significantly with miR‐144 overexpression [105]. Using ROC curve analysis, miR‐144 was found to predict diabetic complications with a sensitivity of 88.7% and specificity of 87.8%, suggesting its potential as a biomarker for assessing diabetes severity.

The suppressor of cytokine signaling 2 (SOCS2) is a key regulator of the inflammatory response and is implicated in the pathogenesis of diabetic nephropathy [106]. Studies in diabetic nephropathy models have identified SOCS2 as a target gene of miR‐144, which in turn can be targeted by cancer susceptibility candidate 2 (CASC2) [107]. Reduced CASC2 expression was observed in both in vivo and in vitro models of diabetic nephropathy. Overexpression of CASC2 mitigated cell apoptosis and the release of inflammatory factors such as IL‐1β, IL‐6, and TNF‐α through the miR‐144/SOCS2 axis, slowing the progression of diabetic nephropathy.

## 13 miR‐144 and Other Diseases

### 13.1 Transfusion‐Related Inflammation

Recently, the proinflammatory role of heme in hemoglobin has been extensively studied. Sun et al. [108] reported that injecting heme into the peritoneal cavity of mice increased leukocyte infiltration and prolonged the resolution time of murine peritonitis. Further investigation revealed that heme could upregulate miR‐144‐3p, which targets and negatively regulates the expression of the lipoxin A4 receptor/formyl peptide receptor 2, a protein with host defense and anti‐inflammatory properties. This study helps to elucidate the role of heme in blood transfusion‐related inflammatory reactions.

### 13.2 Renal Interstitial Fibrosis

Angiopoietin‐like protein 3 (ANGPTL3), a protein with distinct functional domains, is involved in various kidney disorders [109, 110]. Yang et al. [111] observed a significant reduction in ANGPTL3 level and a significant increase in miR‐144‐3p expression in both in vivo and in vitro models of renal interstitial fibrosis. Enhancing ANGPTL3 or inhibiting miR‐144‐3p in experimental models alleviated cellular apoptosis, inflammation, and activation of the PI3K/AKT signaling pathway. This study identified ANGPTL3 as a downstream target of miR‐144‐3p by LRA and suggested that the miR‐144‐3p/ANGPTL3 axis could promote the progression of renal interstitial fibrosis.

### 13.3 Thyroid‐Associated Orbitopathy (TAO)

TAO, also known as Graves’ orbitopathy, is an intractable autoimmune disease, and its pathogenesis is closely related to the inflammatory environment within the orbit [112, 113]. Research by Wei et al. [114] found that the expression of miR‐144‐3p in the exosomes and PBMCs of patients with active TAO was significantly higher than that of healthy controls. The mimics of miR‐144‐3p significantly enhanced the expression of inflammatory molecules including IL‐1β, IL‐6, TNF‐α, and CXCL2.

## 14 The Mechanisms by Which miR‐144 Influences Inflammation

**Figure 2:**
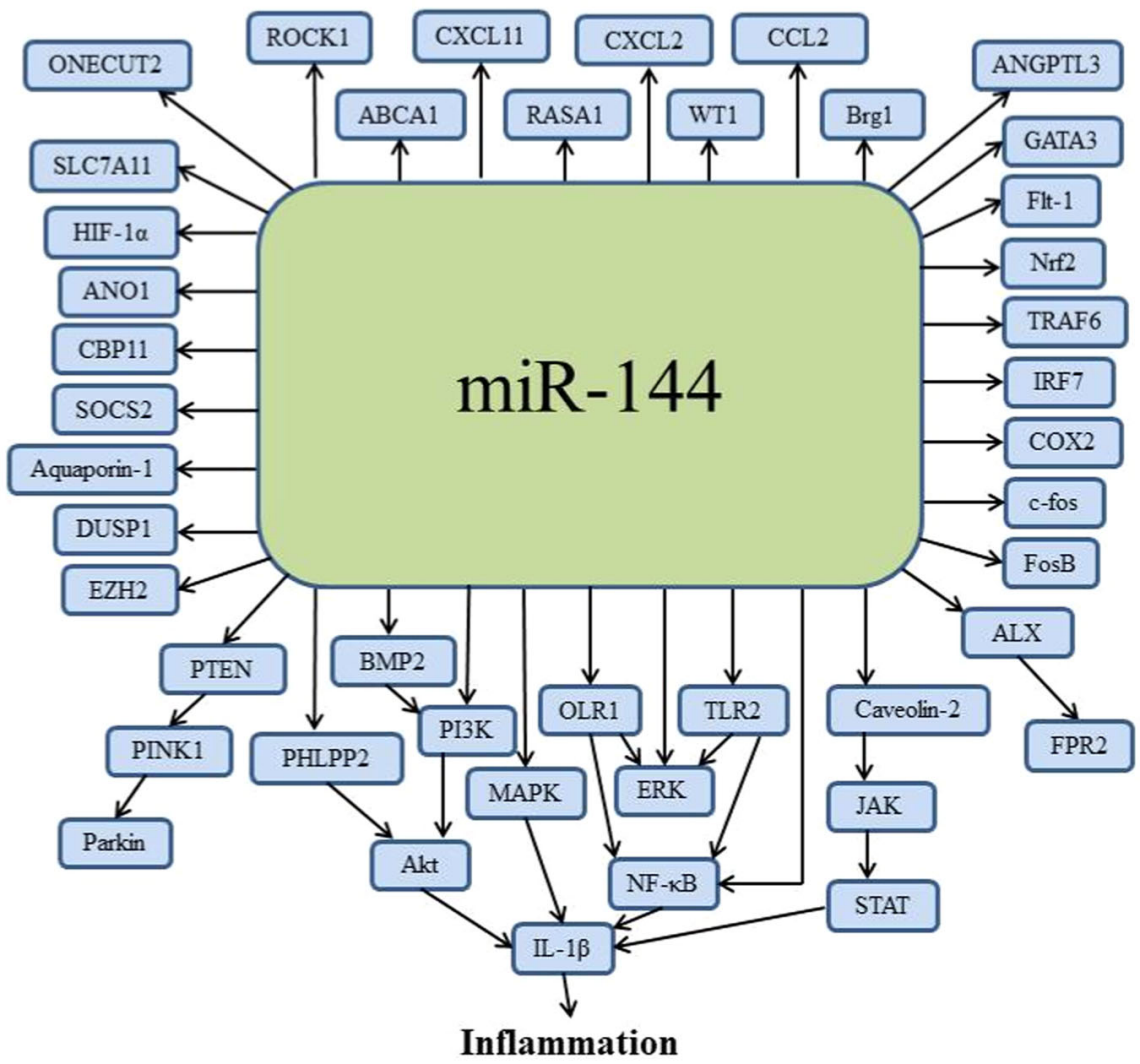
Key factors and pathways regulated by miR‐144 in inflammation. ABCA1, ATP binding cassette transporter A1; Akt, protein kinase B; ALX, lipoxin A4 receptor; ANGPTL3, angiopoietin‐like protein 3; ANO1, Anoctamin 1; BMP2, bone morphogenetic protein 2; Brg1, Brahma‐related gene 1; CBP11, calcium‐binding protein 11; CCL, C‐C motif chemokine ligand; COX2, cyclooxygenase‐2; CXCL, C‐X‐C motif chemokine ligand; DUSP1, dual‐specificity phosphatase 1; ERK, extracellular signal‐regulated kinase; EZH2, enhancer of zeste homolog 2; Flt‐1, FMS‐like tyrosine kinase 1; FPR2, formyl peptide receptor 2; HIF‐1α, hypoxia inducible factor 1 alpha; JAK, Janus kinase; IL, interleukin; IRF7, interferon regulatory factor 7; MAPK, mitogen‐activated protein kinase; NF‐κB, nuclear factor‐kappa B; Nrf2, nuclear factor E2‐related factor 2; OLR1, oxidized low‐density lipoprotein receptor 1; PHLPP2, PH domain leucine‐rich repeat protein phosphatase 2; PI3K, phosphatidylinositol 3‐kinase; PINK1, PTEN‐induced putative kinase 1; PTEN, phosphatase and tensin homolog; RASA1, RAS P21 Protein Activator 1; ROCK1, Rho related kinase‐1; SLC7A11, solute carrier family 7 member 11; SOCS2, suppressor of cytokine signaling 2; STAT, signal transducer and activator of transcription; TLR2, Toll like receptor 2; TRAF6, TNF receptor associated factor 6; WT1, Wilms Tumor 1. Figure 2 *can be considered for online covers*.

Based on the above literature review, we have summarized the expression levels of miR‐144 in inflammatory diseases (Table 1) and its possible mechanisms in inflammation (Figure 2). On the one hand, miR‐144 can directly targeted related signaling pathways, involving the NF‐κB, STAT, MAPK, and Akt pathways. The activation of these pathways will regulate the release of inflammatory mediators such as IL‐1β. Taking the NF‐κB signaling pathway as an example, in resting cells, IκBα, an inhibitor of NF‐κB, interacts with p65 to block nuclear translocation of NF‐κB and anchors NF‐κB in the cytoplasm in an inactive state [115]. When cells are stimulated, miR‐144 can target IκBα to promote its degradation, leading to the activation of p65 and nuclear translocation of NF‐κB [116]. In the nucleus, NF‐κB binds to specific DNA elements to initiate the transcription of proinflammatory genes like IL‐1β. Additionally, miR‐144 can target OLR1 or TLR2 to activate the NF‐κB signaling pathway.

**Table 1:** Summary of expression and mechanism of miR‐144 in inflammatory diseases.

Inflammatory diseases	miR‐144			
	Test samples	Expression	Targets/key factors	References
**Sepsis**	Patient blood	Down	—	[17, 18]
	Mice aorta	Down	HIF‐1α	[19]
	Mice cardiomyocytes	Down	NF‐κB	[4]
	Mice pulmonary endothelial cells	Down	ROCK1	[25]
	Mice kidney	Up	—	[26]
	Mice lung tissue	Up	Caveolin‐2, JAK/STAT	[27]
	Mice alveolar epithelial cells (A549)	Up	Aquaporin‐1	[28]
**Infectious diseases**				
Mycobacterium subspecies respiratory infection	Patient blood	Up	IL‐1β, IL‐6, CXCL2, CCL2	[30]
Pulmonary tuberculosis	Patient blood	Up or down	—	[31, 32]
	Monocyte‐derived macrophages	Down	ERK	[5]
COVID‐19 pneumonia	Patient blood	Down	—	[33]
Influenza virus pneumonia	Mice lung tissue	Up	TRAF6, IRF7	[34]
**Respiratory diseases**				
COPD	Human bronchial epithelial cells (16HBE)	Up	PHLPP2, Akt	[39, 40]
	Patient blood	Up	EZH2	[42]
Lung adenocarcinoma	Patient blood	Down	WT1	[7, 47]
Non‐small cell lung cancer	Cancer tissues and cell lines (A549)	Down	TLR2	[48]
Asthma	Patient lung and blood	Up	GATA3	[51, 53]
**Cardiovascular diseases**				
Myocardial ischemia	Mice cardiac cells (H9c2)	Down	Brg1, ANO1	[55, 60]
Atherosclerosis	Patient blood	Up	ABCA1	[62]
Abdominal aortic aneurysms	Mice abdominal aortic aneurysm tissue	Down	TLR2, OLR1	[65]
**Digestive diseases**				
Nonalcoholic steatohepatitis	Patient blood	Down	—	[66]
	Rat Kupffer cells	Down	TLR2	[67]
Inflammatory bowel disease	Patient blood	Up	—	[70]
	Mice colon tissue	Up	SLC7A11	[72]
Reflux esophagitis	Rat esophageal mucosa	Up	Nrf2	[71]
Colorectal cancer	Patient blood, tumor samples	Down	CXCL11	[72]
**Neuropsychiatric diseases**				
Neuropathic pain	Mice	Down	RASA1	[78]
Cerebral hemorrhage	Mice brain tissue	Down	—	[79]
Cerebral ischemia	Rat brain tissue	Down	—	[80]
Parkinson's disease	Patient brain tissue	Down	NF‐κB	[81]
Aging	Rat microvascular endothelial cells	Up	Nrf2	[86]
Depression	Patient blood	Down	—	[88]
**Arthritis**				
Rheumatoid arthritis	Chondrocytes (N1511)	Up	BMP2 or PTEN	[90, 91]
	Macrophages (THP‐1)	Down	TLR2, NF‐κB	[92]
	Rat	Up	CBP11	[93]
	Mice	Down	HIF‐1α	[94]
Osteoarthritis	Patient synovial tissue	Down	IL‐1β, PI3K, Akt, NF‐κB, MAPK	[6]
	Primary chondrocytes and C28/I2 cells	Up	DUSP1, MAPK	[95]
	Human fibroblast synovial cells	Down	ONECUT2	[96]
**Pregnancy complications**				
Premature labor	Mice amniotic tissue	Up	COX2, c‐fos	[100]
Preeclipsia	Patient placental tissue	Down	FosB, Flt‐1	[104]
**Diabetes**				
Poorly controlled diabetes	Patient blood	Up	—	[105]
Diabetic nephropathy	Human renal mesangial cells	Up	SOCS2	[107]
**Other diseases**				
Blood transfusion‐related inflammation	Mice	Up	ALX/FPR2	[108]
Renal interstitial fibrosis	Human proximal kidney tubuloepithelial HK‐2 cells	Up	ANGPTL3	[111]
Thyroid‐associated orbitopathy	Patient blood	Up	IL‐1β, IL‐6, TNF‐α, CXCL2	[114]

Abbreviations: ABCA1, ATP binding cassette transporter A1; Akt, protein kinase B; ALX/FPR2, lipoxin A4 receptor/formyl peptide receptor 2; ANGPTL3, angiopoietin‐like protein 3; ANO1, Anoctamin 1; BMP2, bone morphogenetic protein 2; Brg1, Brahma‐related gene 1; CBP11, calcium‐binding protein 11; CCL, C‐C motif chemokine ligand; COPD, chronic obstructive pulmonary disease; COVID‐19, Coronavirus disease 2019; COX2, cyclooxygenase‐2; CXCL, C‐X‐C motif chemokine ligand; DUSP1, dual‐specificity phosphatase 1; ERK, extracellular signal‐regulated kinase; EZH2, enhancer of zeste homolog 2; Flt‐1, FMS‐like tyrosine kinase 1; HIF‐1α, hypoxia inducible factor 1 alpha; IL, interleukin; IRF7, interferon regulatory factor 7; JAK/STAT, Janus kinase/signal transducer and activator of transcription; MAPK, mitogen‐activated protein kinase; NF‐κB, nuclear factor‐kappa B; NF‐κB, nuclear factor‐kappa B; Nrf2, nuclear factor E2‐related factor 2; OLR1, oxidized low‐density lipoprotein receptor 1; PHLPP2, PH domain leucine‐rich repeat protein phosphatase 2; PI3K, phosphatidylinositol 3‐kinase; PTEN, phosphatase and tensin homolog; RASA1, RAS P21 Protein Activator 1; ROCK1, Rho related kinase‐1; SLC7A11, solute carrier family 7 member 11; SOCS2, suppressor of cytokine signaling 2; TLR2, Toll like receptor 2; TRAF6, TNF receptor associated factor 6; WT1, Wilms Tumor 1.

On the other hand, miR‐144 can indirectly activate related signaling pathways by targeting upstream key factors of inflammatory cytokines. For example, miR‐144 may regulate transcription factors, cytokine receptors, or other regulatory proteins to indirectly influence inflammation. The activation of these intermediate factors can amplify or inhibit inflammatory signals, affecting the cellular inflammatory responses.

It should be noted that these direct and indirect actions of miR‐144 are not isolated but interact through complex interactions and feedback loops. The expression level of miR‐144 may dynamically adjust its regulation on both direct and indirect pathways based on the inflammatory environment to play different roles in various inflammatory diseases.

## 15 Summary and Perspective

This review comprehensively examines the role of miR‐144 in various inflammatory diseases, highlighting its potential as a key regulatory molecule in the pathogenesis of conditions ranging from sepsis to TAO. The expression patterns of miR‐144 across different studies present a complex picture, with both upregulation and downregulation observed depending on the disease context. Variations in sample sources, study methodologies, individual diversity, and disease states may lead to inconsistencies in research findings. The mechanisms by which miR‐144 exerts its effects are equally diverse, involving direct targeting of key proteins and pathways, such as HIF‐1α, ROCK1, and the NF‐κB signaling cascade, as well as indirect modulation through the regulation of downstream factors like TLR2 and PTEN. The review also underscores the potential of miR‐144 as a biomarker for disease diagnosis and prognosis, such as sepsis, PTB, COPD, IBD, and RA, with its levels in blood and tissue samples correlating with disease severity and patient outcomes.

The findings from the research on miR‐144 offer promising avenues for translating benchtop discoveries into clinical practice. Future clinical studies should focus on validating miR‐144 as a reliable biomarker in large‐scale, multicenter trials, ensuring its utility across diverse populations. Additionally, the therapeutic potential of miR‐144 should be explored through the development of targeted interventions, such as antagomirs or mimics, to modulate its expression in a disease‐specific manner. The safety and efficacy of these interventions will be crucial, particularly in light of the pleiotropic effects of miR‐144. Moreover, understanding the intricate regulatory networks involving miR‐144, including its interactions with other miRNAs and long non‐coding RNAs, will be essential for developing precision medicine approaches. The integration of miR‐144 research with emerging technologies, such as clustered regularly interspaced short palindromic repeats (CRISPR)‐based gene editing and single‐cell RNA sequencing, may provide deeper insights into its role in inflammation and pave the way for novel therapeutic strategies.

## Author Contributions

**Shukun Hong:** conceptualization, data curation, funding acquisition, resources, writing – original draft, writing – review and editing. **Hongye Wang:** conceptualization, data curation, resources, writing – original draft, writing – review and editing. **Lujun Qiao:** investigation, project administration, supervision, writing – review and editing.

## Conflicts of Interest

The authors declare no conflicts of interest.

## Data Availability

All relevant data are within the paper. The data are available from the corresponding author on reasonable request.
